# Supplementary material for: Move Well, Feel Good: Feasibility and acceptability of a school-based motor competence intervention to promote positive mental health
Source: PLoS One. 2024 Jun 11;19(6):e0303033. doi: 10.1371/journal.pone.0303033 (PMC11166299; doi:10.1371/journal.pone.0303033)
Supplement: S1 File — (PDF) [file pone.0303033.s001.pdf]

Move Well, Feel Good PE lesson plan (Y5)

**Block number (& FMS theme):** 3 (locomotor skills)	**Lesson number:** 7

Lesson focus

By the end of the lesson the pupils will have learned:

1. To develop and practice jumping skills
2. To practice combining locomotor and stability skills with proficiency
3. To apply locomotor skills to situations requiring changes or direction and level
4. To demonstrate understanding and contextual application of communication, cooperation, empathy, encouragement, resilience, self-improvement, determination

Resources and equipment

- Gym mats
- Marker cones

Warm-up

1. Lily Pads and Bull Rushes game: see resource card **B3_L7_WU1**

Skills Introduction & development

1. Frog Hopping individual activity following teacher demonstration with key teaching points: see resource card **B3_L7_I1**
2. Pogo Jumping individual activity following teacher demonstration with key teaching points: see resource card **B3_L7_I2**
3. Frog, Pogo, Hop Tag group activity: see resource card **B3_L7_I3**
4. Running individual activity following teacher demonstration with key teaching points: see resource card **B3_L7_I4**

Skills Progression

1. Partner Tag pairs activity: see resource card **B3_L7_P1**
2. Foxes and Bunnies individual activity and game: see resource card **B3_L7_P2**

Skills Introduction & development 2

1. Crawling Soldier: individual activity following teacher demonstration with key teaching points: see resource card **B3_L7_I5**

Skills Application (depending on pupils' progress use one or both activities)

1. Trains and Tunnels pairs then whole group activity: see resource card **B3_L7_A1**

Finish-up

1. Opposites cool-down activity: see resource card **B3_L7_F1**

Teacher-led Q&A asking pupils to recap what they have learned, focusing on

2. Key points related to performing the different types of jumps
3. Examples of working cooperatively and how they did this effectively
4. Examples of other psycho-social skills they developed during the lesson

Signposting to at-home activities and digital resources

Pupils encouraged to practice their locomotor skills at home alone and with a sibling or parent guided by the following short video resources (accessed via QR codes)

- Crawling solider in different directions
- Holding a 'tunnel' position
