# Supplementary material for: Move Well, Feel Good: Feasibility and acceptability of a school-based motor competence intervention to promote positive mental health
Source: PLoS One. 2024 Jun 11;19(6):e0303033. doi: 10.1371/journal.pone.0303033 (PMC11166299; doi:10.1371/journal.pone.0303033)
Supplement: S2 File — (PDF) [file pone.0303033.s002.pdf]

Week 7

Crawling soldiers in different directions

1. Lie on your tummy.

2. Crawl along on your tummy by pulling with your arms and pushing on your legs, with arms and legs working in opposition to each other.

3. Bend and straighten your arms and legs to help you move.

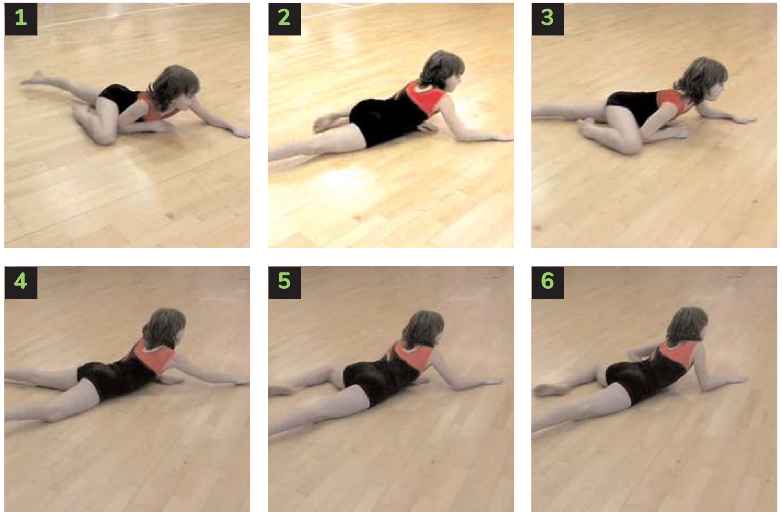

Challenge

- Try moving sideways
- Try moving backwards
- Try moving with your arms only

Acknowledgements: Resource adapted from the original produced by Sport Wales (www.citbag.sport.wales/en)

Week 7

Holding a 'tunnel' position

1. Hands and feet on the floor showing a wide shape.

2. Arms and legs straight.

3. Hold the position.

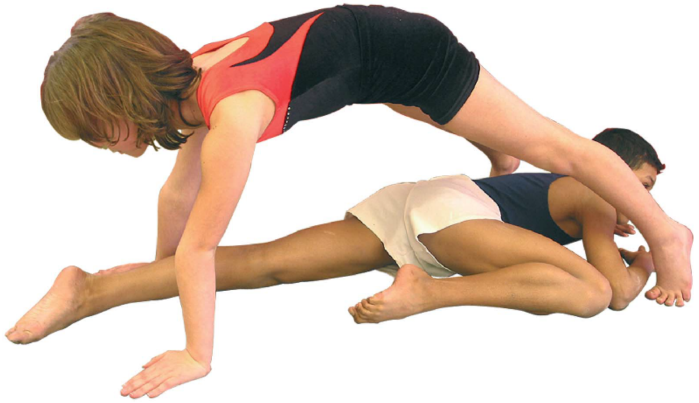

Challenge

- Make a tunnel as high and as wide as you can.
- Make a tunnel facing the ceiling.

Acknowledgements: Resource adapted from the original produced by Sport Wales (www.citbag.sport.wales/en)
