# Supplementary material for: Move Well, Feel Good: Feasibility and acceptability of a school-based motor competence intervention to promote positive mental health
Source: PLoS One. 2024 Jun 11;19(6):e0303033. doi: 10.1371/journal.pone.0303033 (PMC11166299; doi:10.1371/journal.pone.0303033)
Supplement: S3 File — (PDF) [file pone.0303033.s003.pdf]

S3. Detailed description of feasibility outcome measure methods and administration

Each week the PE teachers were asked to 1) complete a delivery log which detailed whether the MWFG PE lesson had been taught, its duration, number of children present, and whether the at-home skill snacks were suggested to the class, and 2) complete a weekly delivery log which asked through which academic subject the MWFG components were taught during classroom lessons and which aspects of motor competence and/or psychosocial development were incorporated. Short feasibility and acceptability surveys were completed by PE teachers and class teachers at the end of the intervention. Both included statements followed by a 1-5 Likert scale corresponding to the responses ‘strongly disagree’ [1], ‘disagree’, ‘unsure’, ‘agree’, and strongly agree’ [5]. The PE teacher survey included two statements which related to the MWFG lesson plans and resources being straightforward to follow and use, and the MWFG lessons being pitched at the appropriate levels for the children’s abilities. The class teacher survey included four statements. Two related to the intervention (‘the MWFG programme and resources were straightforward to follow and use’; ‘the MWFG programme was engaging and interesting for the children’), and two related to the data collection methods (‘the methods used to collect the data worked well’; ‘the methods used to collect the data were interesting and engaging for the children’). A survey was also completed by the children using a 5-point Likert scale but with simplified language and sad to smiley face emojis which were coloured red through green. The emojis corresponded to the responses ‘I don’t agree at all’ (red very sad face emoji), ‘I don’t agree’, ‘I’m not sure’, ‘I agree’, and ‘I really agree’ (green very happy face emoji). Children responded to three statements related to the MWFG PE lessons being enjoyable, learning new skills during the MWFG PE lessons, and enjoyment of the data collection sessions.

One focus group was conducted in each school to gain children's views on the intervention activities and data collection methods. These comprised of three boys and three girls who were chosen at random by the class teachers. Two participatory visual methods were used, one produced by the children (write, draw, show tell (1)) and one provided by the research team (photo elicitation). The write, draw, show, tell activity icebreaker task engaged children in the focus group and acted as a stimulus for the children to engage. The children were asked to write or draw the activities that were most memorable to them throughout the MWFG programme. They were then asked to describe what they had drawn and explain why. Next, photos of the data collection methods were used to prompt children to discuss their perceptions of the data collection sessions. All teachers were invited to take part in semi-structured interviews to gain a deeper understanding of the programme's feasibility and acceptability. Class teachers and PE teachers from three schools were interviewed, with the remainder unable to engage due to limited availability. Focus groups and semi-structured interviews were recorded using a digital audio recorder and were transcribed verbatim. All feasibility outcome data were collected at T1.

References

1. Noonan RJ, Boddy LM, Fairclough SJ, Knowles ZR. Write, draw, show, and tell: a child-centred dual methodology to explore perceptions of out-of-school physical activity. BMC Public Health. 2016;16(1):1-19.
