# Supplementary material for: Move Well, Feel Good: Feasibility and acceptability of a school-based motor competence intervention to promote positive mental health
Source: PLoS One. 2024 Jun 11;19(6):e0303033. doi: 10.1371/journal.pone.0303033 (PMC11166299; doi:10.1371/journal.pone.0303033)
Supplement: S5 File — (PDF) [file pone.0303033.s005.pdf]

Feasibility of a co-produced primary school-based intervention to improve children's motor competence and mental health and wellbeing (shortened title: Motor competence and mental health)

Principal Investigator: Prof Stuart Fairclough; email: stuart.fairclough@edgehill.ac.uk; tel: 01695584143; Dept. Sport & Physical Activity, St Helens Road, Ormskirk, Lancs L39 4QP; https://research.edgehill.ac.uk/en/persons/stuart-fairclough
Research Office contact: Jenny Lawson (lawsonj@edgehill.ac.uk)

Applicants
Prof Stuart Fairclough and Dr Richard Tyler (tylerr@edgehill.ac.uk) are at Edge Hill University. Prof Zoe Knowles (z.r.knowles@ljmu.ac.uk), Dr Lawrence Foweather (l.foweather@ljmu.ac.uk), Dr Lynne Boddy (l.m.boddy@ljmu.ac.uk), and Dr Emma Ashworth (e.l.ashworth@ljmu.ac.uk) are at Liverpool John Moores University.

Scientific abstract
Children's mental health has been negatively affected during the COVID-19 lockdown restrictions. According to the Elaborated Environmental Stress Hypothesis, anxiety and depression in children are associated with poor motor competence, and these associations may be mediated by social support and self-perceptions. Improving children's motor competence may therefore be a mechanism for promoting mental health through psychosocial factors. There is some evidence that schools are effective settings to improve mental health outcomes through motor skill interventions. Thus, we aim to co-produce and evaluate the feasibility of a school-based motor competence and mental health intervention. Six primary schools (four intervention, two control) from low socioeconomic status locations will be recruited. Child, teacher, school leader, and physical activity deliverer stakeholder groups will take part in an intervention co-production process to develop a 12-week motor competence and mental health intervention. Focus groups, interviews, observations, questionnaires, and motor competence assessments will be used to gather data on the feasibility and acceptability of the intervention to determine whether it should be subsequently developed into a pilot cluster RCT. Pre-post intervention changes in motor competence and mental health and the influence of psychosocial factors will also be explored.

Lay summary
Children's motor skill ability is linked to mental health problems such as anxiety and depression. The COVID-19 lockdown restrictions have negatively affected children's mental health and reduced their participation in physical activity, which is vital for motor skill development. As school-based programmes are promising ways to tackle declines in motor skills and mental health, we will work with children, teachers, school leaders, and physical activity specialists to develop a primary school intervention to improve children's motor skills and mental health. We will use different research methods to see how feasible and acceptable the intervention and our research approaches are. We will also measure the children's motor skills and mental health to see whether these change during the intervention, and how they relate to other factors like physical activity, and support from others. We will share our findings with children, teachers, physical activity providers, public health officials, and the academic community.

Theoretical and scientific rationale
The COVID-19 pandemic social distancing measures have resulted in unprecedented, enforced changes to people's routines and lifestyles. Children have been particularly affected, through school closures and online home learning, the ceasing of organised sports, and restrictions on face-to-face social interactions. There is evidence that lockdown restrictions have negatively affected children's mental health and wellbeing (1), with those from lower socioeconomic

status families disproportionately affected (2, 3). Furthermore, lockdown restrictions have driven increases in digital screen use and decreases in physical activity, and particularly structured activities (e.g., physical education lessons, sport participation (4, 5)), which are essential for development of motor competence (6) (i.e., the degree to which a child performs goal-directed movements in a coordinated, accurate, and relatively error-free manner (7)). Thus, reduced physical activity during the pandemic is likely to be reflected by attenuation of motor competence or slowing of motor competence development (8). Poor motor competence is associated with inhibited psychosocial development, including internalising problems such as anxiety and depression (9); these associations may be mediated by limited social support mechanisms (9, 10) and low self-perceptions (11, 12). Improving children's motor competence may therefore be a mechanism for promoting children's mental health through enhancing aspects of psychosocial development.

The inter-relationships between children's motor competence, mental health, and their mediators are described in the Elaborated Environmental Stress Hypothesis (EESH) (13). Empirical support exists for the EESH (14), which posits that poor motor competence predisposes children to internalising mental health problems (e.g., depression, anxiety) via interactions with environmental stressors such as low self-esteem, low social support, physical inactivity, overweight, etc. These stressors can in turn be 'buffered' by social and personal resources such as parental support and perceived competence (14). Previous studies in community samples have reported negative associations between motor competence, internalising problems and other psychosocial outcomes described in the EESH (10, 15). This proposal will be conceptually underpinned by the EESH and the 'buffering' components will be targeted as underlying characteristics of a proposed intervention.

In 2018 applicants SF and RT were awarded TWF grant #1669-3509. They found that low levels of motor competence were prevalent in 33% of the sampled 9-10 year old children. Compared to peers with superior motor competence, these less skilled children had more internalising mental health problems, poorer academic performance, and unfavourable physical activity levels (manuscript in review). These data and published evidence highlight a need for intervention strategies in primary school children to address the established low levels of motor competence and mental health and wellbeing which have declined further as a consequence of the COVID-19 lockdown measures (1, 8). Schools are suitable settings for the promotion of child health and wellbeing (16). Furthermore, primary school motor competence interventions can be efficacious for improving motor skills (17, 18) and there is some limited evidence that they can also enhance mental health and wellbeing (19, 20). However, no such studies involving mainstream school children without movement difficulties have been undertaken in the UK.

School stakeholders are more likely to 'buy-in' to interventions that are co-produced with children and teachers, and that align to statutory Personal, Social, Health and Economic Education curriculum guidance (21, 22). Co-production provides opportunities to participate in intervention development processes (23), thereby ensuring that the specific needs of stakeholders are targeted. The shared stakeholder ownership of the process provides a context-sensitive basis for an acceptable intervention with increased likelihood of it being effectively implemented and resulting in positive outcomes (21). Therefore, working with children, teachers, school leaders, and physical activity deliverers our aim is to co-produce and evaluate the feasibility of a primary school intervention to improve children's motor competence and mental health and wellbeing.

Feasibility of a co-produced primary school-based intervention to improve children's motor competence and mental health and wellbeing (shortened title: Motor competence and mental health)

Practical impact of the project

Please see section 'Expected impact of the project' on p. 6.

Research design

Participants would be Year 4 and Year 5 (age 8-10 years) children from one or two-form entry mainstream primary schools in West Lancashire who are physically able to participate in PE lessons. Schools will be located in low socioeconomic status (SES) areas based on school postcode-linked Indices of Multiple Deprivation (IMD; deciles 1-3)) (24) and >18% free-school meal (FSM) eligibility. Schools meeting these criteria and with at least 25 pupils per class, will be approached in the order produced by a random number generator until six agree to participate. After this they will be randomised to the intervention (4 schools) or usual practice control arms (2 schools). Using progression criteria Red/Stop upper limit and Green/Go lower limit reference tables provided by Lewis et al. (25) we estimated an intervention group sample size for child recruitment. Assuming an alpha level of 0.05 and 90% power to reject being in the Red zone if the Green zone holds true, the minimum required sample size is n=46. We propose to recruit up to 200 children to the intervention group (and 50 to the control group), which is significantly more than the estimates from the power calculation, and than the larger end of the sample size scale (n=100) typically observed in health behavioural pilot and feasibility studies (26). This will ensure a representative range of responses to the measures and engagement in the feasibility intervention. Thus, we believe that the planned sample size is sufficient to meet the study aim. Our recruitment strategy will include school and child participation incentives (e.g., vouchers).

<u>Intervention co-production.</u> Intervention co-production will involve children, teachers, school leaders, and physical activity deliverers from West Lancashire Sport Partnership (WLSP). These stakeholders will work in single and multiple stakeholder groups through a 6-stage process facilitated by the researchers. These stages align to the Double Diamond Design Approach (DDDA) by employing divergent and convergent thinking processes as stakeholders discover, define, develop, and deliver a solution to the 'problems' of how best to improve children's motor competence and mental health, and how best to facilitate real-world implementation in school contexts (Table 1) (27). The intention is that the DDDA will enable stakeholder groups to reach consensus on the components and content of a 12-week (i.e., 1 school term) school-based motor competence and mental health intervention, underpinned by social support and perceived competence as key elements of the EESH. To engage the children in the process without them feeling inhibited in expressing their thoughts in the presence of the adults that regularly teach them, they will operate in a single stakeholder group in Stages 1, 2 (after which their discussion points will be shared in Stage 3), 4, and 6. Following this co-production process the 12-week feasibility intervention study will be implemented.

<u>Study outcomes.</u> This is a feasibility study which asks whether the planned intervention can be done, whether it should be developed into a pilot cluster RCT, and if so, how (28). Given the scarcity of previous school-based interventions to simultaneously improve motor competence and mental health outcomes in community samples (14), a number of uncertainties relating to the conduct of a larger trial need assessing (28, 29). These uncertainties represent the primary outcomes and broadly relate to eligibility and recruitment, deliverer capability and training, practicalities of implementation, acceptability of data collection procedures, intervention adherence, and data attrition. These feasibility outcomes will be measured using qualitative and quantitative methods. In each school we will use focus groups and other appropriate participatory approaches to gain children's views on the intervention activities and data

collection methods. Semi-structured teacher and WLSP deliverer interviews will examine intervention acceptability and implementation experiences. The quantitative feasibility outcomes will be evaluated using *a priori* traffic light progression criteria (i.e., green: continue to pilot trial, amber: further discussion and changes needed, red: do not proceed to pilot trial; Table 2) (30).

Thinking processes	Stage	Tasks	Single (SS) or multiple stakeholder (MS) groups
	1	Information workshops on child motor competence (MC) and mental health (MH) in the context of the EESH. These will ensure minimum required knowledge and understanding to engage in subsequent stages	SS
Divergent	2	Discussion of MC and MH in school context and identify what can be done in schools to improve MC and MH	SS then repeat in MS
Convergent	3	Draft intervention component ideas for improving MC and MH over a school term within curriculum and non-curriculum time	MS
Divergent	4	Each MS group presents draft ideas for discussion and feedback from other MS groups	MS
Convergent	5	Each MS group refines their ideas based on Stage 4 and re-presents to other MS groups (e.g., as a visual model)	MS
Convergent	6	Individual participants vote on the Stage 5 models to reach consensus on the preferred co-produced intervention	Individuals

Table 1. Six-stage co-production processes incorporating DDDA in stages 2 to 6

Progression criteria	Red (stop)	Amber (discuss and amend)	Green (go)
School recruitment (targeting N=6)	≤50% of target number	50-90% of target number	≥90% of target number
Child participant recruitment (targeting N≥200)	<20% of eligible children	20-74% of eligible children	≥75% of eligible children
Deliverer recruitment	≥1 teacher and ≥1 WLSP deliverer in <50% of schools	≥1 teacher and ≥1 WLSP deliverer in ≥50%-75% of schools	≥1 teacher and ≥1 WLSP deliverer per school
Intervention dose	<40% of scheduled sessions delivered/week	40-79% of scheduled sessions delivered/week	≥80% of scheduled sessions delivered/week
Intervention adherence	<40% of recruited children attend ≥75% of curriculum lessons	40-69% of recruited children attend ≥75% of curriculum lessons	≥70% of recruited children attend ≥75% of curriculum lessons
Acceptability of intervention	<50% of teachers and children	50-79% of teachers and children	≥80% of teachers and children
Acceptability of data collection methods	<50% of teachers and children	50-79% of teachers and children	≥80% of teachers and children
Secondary outcome data collected at baseline	<50% of children	50-74% of children	≥75% of children
Follow-up secondary outcome data attrition	>40%	26-40%	≤25%

Table 2. Traffic light progression criteria

The secondary outcomes specified below relate to key elements of the EESH (13, 14). Multiple measures will be trialled to assess internalising difficulties and wellbeing as there is uncertainty as to what methods are most appropriate and acceptable. Thus, we will assess internalising difficulties using the Strengths and Difficulties Questionnaire (31) and Me and My Feelings questionnaire (32), and wellbeing through the Stirling Children's Wellbeing Scale (33) and

KIDSCREEN-10 questionnaire (34). To limit participant burden, just one from each pair of these secondary outcome measures will be used in separate sub-samples of children from different intervention and control schools. Motor competence will be measured by the Dragon Challenge dynamic motor competence assessment (35), supplemented by the parent-completed Developmental Coordination Disorder (DCD) Questionnaire (36). Psychosocial stressors and buffers relevant to the EESH and the respective measurement instruments include self-concept (Self-Perception Profile for Children (37)), social support for physical activity (38), peer support (Student Resilience Survey peer connection subscale (39)), social skills (Strengths and Difficulties Questionnaire prosocial behaviour subscale (40)), academic achievement (National Curriculum attainment levels), movement behaviours (24-h wrist accelerometery), and sport participation (Sport England Active Lives Survey for Children and Young People selected questions (41)). As part of our determination of feasibility we will record the time needed to complete the questionnaires and the completion rates. Further, we will examine the responsiveness of these measurement tools to detect changes in the outcomes as a result of the intervention. Data for potential moderators of the relationships between motor competence and internalising problems such as sex, SES (parent education level), Special Educational Needs/Additional Learning Support status, and weight status (42) will also be collected.

Methods of analysis. Thematic analysis (semantic and inductive) of the qualitative data will identify key themes and patterns regarding participants' experiences and perceptions of the intervention. As previously mentioned, although the study is not powered by a formal sample size calculation we do acknowledge the need to ensure we have sufficient participants to provide statistical point estimates and determine parameter variability, which would provide valuable information for a subsequent trial sample size calculation (43). We would analyse these quantitative outcomes descriptively and then with linear mixed models.

Rationale for the design and methods. Mixed-methods approaches will allow us to fully integrate participants' views and perceptions to inform and provide essential context to the quantitative outcomes. We have used these approaches effectively in previous work (44-46).

Ethical issues
The project requires an ethics application to EHU's Science Research Ethics Committee. Ethical approval would be obtained before the project start date and period of active funding.

References

1. Wunsch K, Nigg C, Niessner C, Schmidt SCE, Oriwol D, Hanssen-Doose A, et al. The Impact of COVID-19 on the Interrelation of Physical Activity, Screen Time and Health-Related Quality of Life in Children and Adolescents in Germany: Results of the Motorik-Modul Study. Children. 2021;8(2):98.
2. Ravens-Sieberer U, Kaman A, Erhart M, Devine J, Schlack R, Otto C. Impact of the COVID-19 pandemic on quality of life and mental health in children and adolescents in Germany. European Child & Adolescent Psychiatry. 2021.
3. Raw J, Waite P, Pearcey S, Creswell C, Shum A, Patalay P. Examining changes in parent-reported child and adolescent mental health throughout the UK's first COVID-19 national lockdown (preprint). PsyArXix. 2021;https://doi.org/10.31234/osf.io/exktj.

4. England S. Active Lives Children and Young People Survey Coronavirus (Covid-19) Report. London: Sport England; 2021.
5. Moore SA, Faulkner G, Rhodes RE, Brussoni M, Chulak-Bozzer T, Ferguson LJ, et al. Impact of the COVID-19 virus outbreak on movement and play behaviours of Canadian children and youth: a national survey. International Journal of Behavioral Nutrition and Physical Activity. 2020;17(1):85.
6. Tyler R, Mackintosh KA, Foweather L, Edwards LC, Stratton G. Youth motor competence promotion model: a quantitative investigation into modifiable factors. Journal of Science and Medicine in Sport. 2020;23(10):955-61.
7. Foweather L, Rudd JR. Fundamental movement skill interventions. In: Brusseau TA, Fairclough SJ, Lubans DR, editors. The Routledge Handbook of Youth Physical Activity. London: Routledge; 2020. p. 715-37.
8. Pombo A, Luz C, de Sá C, Rodrigues LP, Cordovil R. Effects of the COVID-19 Lockdown on Portuguese Children's Motor Competence. Children. 2021;8(3):199.
9. Mancini VO, Rigoli D, Heritage B, Roberts LD, Piek JP. The Relationship between Motor Skills, Perceived Social Support, and Internalizing Problems in a Community Adolescent Sample. Frontiers in Psychology. 2016;7(543).
10. Wilson A, Piek JP, Kane R. The Mediating Role of Social Skills in the Relationship between Motor Ability and Internalizing Symptoms in Pre-primary Children. Infant and Child Development. 2013;22(2):151-64.
11. Rigoli D, Piek JP, Kane R. Motor Coordination and Psychosocial Correlates in a Normative Adolescent Sample. Pediatrics. 2012;129(4):e892-e900.
12. Viholainen H, Aro T, Purtsi J, Tolvanen A, Cantell M. Adolescents' school-related self-concept mediates motor skills and psychosocial well-being. British Journal of Educational Psychology. 2014;84(2):268-80.
13. Cairney J, Rigoli D, Piek J. Developmental coordination disorder and internalizing problems in children: The environmental stress hypothesis elaborated. Developmental Review. 2013;33(3):224-38.
14. Mancini VO, Rigoli D, Cairney J, Roberts LD, Piek JP. The Elaborated Environmental Stress Hypothesis as a Framework for Understanding the Association Between Motor Skills and Internalizing Problems: A Mini-Review. Frontiers in Psychology. 2016;7(239).
15. Poole KL, Schmidt LA, Missiuna C, Saigal S, Boyle MH, Van Lieshout RJ. Motor coordination and mental health in extremely low birth weight survivors during the first four decades of life. Research in Developmental Disabilities. 2015;43-44:87-96.
16. Langford R, Bonell CP, Jones HE, Pouliou T, Murphy SM, Waters E, et al. The WHO Health Promoting School framework for improving the health and well-being of students and their academic achievement. Cochrane Database Syst Rev. 2014(4):CD008958.
17. Eddy LH, Wood ML, Shire KA, Bingham DD, Bonnick E, Creaser A, et al. A systematic review of randomised and case-controlled trials investigating the effectiveness of school-based motor-skill interventions in 3-12-year-old children. Child: Care, Health and Development. 2019;45:773-90.
18. Engel AC, Broderick CR, van Doorn N, Hardy LL, Parmenter BJ. Exploring the Relationship Between Fundamental Motor Skill Interventions and Physical Activity Levels in Children: A Systematic Review and Meta-analysis. Sports Medicine. 2018.
19. Piek JP, Kane R, Rigoli D, McLaren S, Roberts CM, Rooney R, et al. Does the Animal Fun program improve social-emotional and behavioural outcomes in children aged 4–6 years? Human Movement Science. 2015;43:155-63.

20. Yu JJ, Burnett AF, Sit CH. Motor Skill Interventions in Children With Developmental Coordination Disorder: A Systematic Review and Meta-Analysis. Archives of Physical Medicine and Rehabilitation. 2018;99(10):2076-99.
21. Craig P, Dieppe P, Macintyre S, Michie S, Nazareth I, Petticrew M. Developing and evaluating complex interventions: the new Medical Research Council guidance. BMJ. 2008;337:a1655.
22. Department for Education. Guidance. Personal, social, health, and economic (PSHE) education 2020 [Available from: https://www.gov.uk/government/publications/personal-social-health-and-economic-education-pshe/personal-social-health-and-economic-pshe-education.
23. Reed H, Couturiaux D, Davis M, Edwards A, Janes E, Kim HS, et al. Co-production as an Emerging Methodology for Developing School-Based Health Interventions with Students Aged 11–16: Systematic Review of Intervention Types, Theories and Processes and Thematic Synthesis of Stakeholders' Experiences. Prevention Science. 2020.
24. English Indices of Deprivation 2019 [Internet]. 2019 [cited 9 June 2020]. Available from: http://imd-by-postcode.opendatacommunities.org/imd/2019.
25. Lewis M, Bromley K, Sutton CJ, McCray G, Myers HL, Lancaster GA. Determining sample size for progression criteria for pragmatic pilot RCTs: the hypothesis test strikes back! Pilot and Feasibility Studies. 2021;7(1):40.
26. Beets MW, von Klinggraeff L, Weaver RG, Armstrong B, Burkart S. Small studies, big decisions: the role of pilot/feasibility studies in incremental science and premature scale-up of behavioral interventions. Pilot and Feasibility Studies. 2021;7(1):173.
27. Daly-Smith A, Quarmby T, Archbold VSJ, Corrigan N, Wilson D, Resaland GK, et al. Using a multi-stakeholder experience-based design process to co-develop the Creating Active Schools Framework. International Journal of Behavioral Nutrition and Physical Activity. 2020;17(1):13.
28. Eldridge SM, Lancaster GA, Campbell MJ, Thabane L, Hopewell S, Coleman CL, et al. Defining Feasibility and Pilot Studies in Preparation for Randomised Controlled Trials: Development of a Conceptual Framework. PLOS ONE. 2016;11(3):e0150205.
29. Research NIfH. Guidance on applying for feasibility studies 2021 [Available from: https://www.nihr.ac.uk/documents/guidance-on-applying-for-feasibility-studies/20474.
30. Avery KNL, Williamson PR, Gamble C, O'Connell Francischetto E, Metcalfe C, Davidson P, et al. Informing efficient randomised controlled trials: exploration of challenges in developing progression criteria for internal pilot studies. BMJ Open. 2017;7(2):e013537.
31. Goodman A, Lamping DL, Ploubidis GB. When to Use Broader Internalising and Externalising Subscales Instead of the Hypothesised Five Subscales on the Strengths and Difficulties Questionnaire (SDQ): Data from British Parents, Teachers and Children. J Abnorm Child Psychol. 2010;38(8):1179-91.
32. Deighton J, Tymms P, Vostanis P, Belsky J, Fonagy P, Brown A, et al. The Development of a School-Based Measure of Child Mental Health. Journal of Psychoeducational Assessment. 2013;31(3):247-57.
33. Liddle I, Carter GFA. Emotional and psychological well-being in children: the development and validation of the Stirling Children's Well-being Scale. Educational Psychology in Practice. 2015;31(2):174-85.
34. Ravens-Sieberer U, Erhart M, Rajmil L, Herdman M, Auquier P, Bruil J, et al. Reliability, construct and criterion validity of the KIDSCREEN-10 score: a short measure for children and adolescents' well-being and health-related quality of life. Quality of Life Research. 2010;19(10):1487-500.

35. Tyler R, Foweather L, Mackintosh KA, Stratton G. A Dynamic Assessment of Children's Physical Competence: The Dragon Challenge. Med Sci Sports Exerc. 2018;50(12):2474-87.
36. Wilson BN, Crawford SG, Green D, Roberts G, Aylott A, Kaplan BJ. Psychometric Properties of the Revised Developmental Coordination Disorder Questionnaire. Physical & Occupational Therapy In Pediatrics. 2009;29(2):182-202.
37. Harter S. Self-Perception Profile for Children: Manual and Questionnaires (Grades 3-8). Denver, Colarado: University of Denver; 2012.
38. Ward DS, Saunders RP, Pate RR. Physical Activity Interventions in Children and Adolescents. Champaign, IL: Human Kinetics; 2007.
39. Lereya ST, Humphrey N, Patalay P, Wolpert M, Böhnke JR, Macdougall A, et al. The student resilience survey: psychometric validation and associations with mental health. Child and Adolescent Psychiatry and Mental Health. 2016;10(1):44.
40. Goodman R. Psychometric properties of the strengths and difficulties questionnaire. J Am Acad Child Adolesc Psychiatry. 2001;40(1):1337-45.
41. Sport England. Active Lives Children and Young People Survey. Academic year 2019/20. London: Sport England; 2021.
42. Cole T, Bellizzi M, Flegal K, Dietz W. Establishing a standard definition for child overweight and obesity worldwide: international survey. BMJ. 2000;320:1240 - 3.
43. Lancaster GA, Dodd S, Williamson PR. Design and analysis of pilot studies: recommendations for good practice. Journal of Evaluation in Clinical Practice. 2004;10(2):307-12.
44. Mackintosh K, Knowles Z, Ridgers ND, Fairclough SJ. Using formative research to develop CHANGE!: a curriculum-based physical activity promoting intervention. BMC Public Health. 2011;11(1):831.
45. Taylor SL, Noonan RJ, Knowles ZR, McGrane B, Curry WB, Fairclough SJ. Acceptability and Feasibility of Single-Component Primary School Physical Activity Interventions to Inform the AS:Sk Project. Children. 2018;5(12):171.
46. Owen M, Kerner C, Taylor S, Noonan R, Newson L, Kosteli M-C, et al. The Feasibility and Acceptability of The Girls Peer Activity (G-PACT) Peer-led Mentoring Intervention. Children. 2018;5(9):128.
