# Supplementary material for: Move Well, Feel Good: Feasibility and acceptability of a school-based motor competence intervention to promote positive mental health
Source: PLoS One. 2024 Jun 11;19(6):e0303033. doi: 10.1371/journal.pone.0303033 (PMC11166299; doi:10.1371/journal.pone.0303033)
Supplement: S6 File — (PDF) [file pone.0303033.s006.pdf]

Distribution of total CAMSA scores by physical competence domain categories

	CAMSA category and time point							
	T0 Beginning (%)	T1 Beginning (%)	T0 Progressing (%)	T1 Progressing (%)	T0 Achieving (%)	T1 Achieving (%)	T0 Excelling (%)	T1 Excelling (%)
Boys (n=52)	5.8	1.9	7.7	1.9	9.6	1.9	76.9	94.2
Girls (n=56)	3.6	0	16.1	7.1	3.6	5.4	76.8	87.5
